# Dose modification dynamics of ponatinib in patients with chronic-phase chronic myeloid leukemia (CP-CML) from the PACE and OPTIC trials

**DOI:** 10.1038/s41375-024-02159-0

**Published:** 2024-01-29

**Authors:** Elias Jabbour, Jane Apperley, Jorge Cortes, Delphine Rea, Michael Deininger, Elisabetta Abruzzese, Charles Chuah, Daniel J. DeAngelo, Andreas Hochhaus, Jeffrey H. Lipton, Michael Mauro, Franck Nicolini, Javier Pinilla-Ibarz, Gianantonio Rosti, Philippe Rousselot, Neil P. Shah, Moshe Talpaz, Alexander Vorog, Xiaowei Ren, Hagop Kantarjian

**Affiliations:** 1 The University of Texas MD Anderson Cancer Center, Houston, TX USA; 2 Imperial College London, London, UK; 3 Georgia Cancer Center, Augusta, GA USA; 4 Hôpital Saint-Louis, Paris, France; 5 Versiti Blood Research Institute, Medical College of Wisconsin, Milwaukee, WI USA; 6 S. Eugenio Hospital, Tor Vergata University, Rome, Italy; 7 Singapore General Hospital, National Cancer Centre Singapore, Duke-NUS Medical School, Singapore, Singapore; 8 Dana-Farber Cancer Institute, Boston, MA USA; 9 Universitätsklinikum Jena, Jena, Germany; 10 Princess Margaret Cancer Centre, Toronto, ON Canada; 11 Memorial Sloan Kettering, New York, NY USA; 12 Centre Léon Bérard, Lyon, France; 13 H. Lee Moffitt Cancer Center & Research Institute, Tampa, FL USA; 14 IRST/IRCCS “Dino Amadori”, Meldola (FC), Italy; 15 Hospital Mignot University de Versailles Saint-Quentin-en-Yvelines, Paris, France; 16 University of California San Francisco, San Francisco, CA USA; 17 Comprehensive Cancer Center, University of Michigan, Ann Arbor, MI USA; 18 Takeda Development Center Americas, Inc., Lexington, MA USA

**Keywords:** Chronic myeloid leukaemia, Phase II trials

## Abstract

Ponatinib, the only approved all known-BCR::ABL1 inhibitor, is a third-generation tyrosine-kinase inhibitor (TKI) designed to inhibit BCR::ABL1 with or without any single resistance mutation, including T315I, and induced robust and durable responses at 45 mg/day in patients with CP-CML resistant to second-generation TKIs in the PACE trial. However, cardiovascular toxicities, including arterial occlusive events (AOEs), have emerged as treatment-related AEs within this class of TKIs. The OPTIC trial evaluated the efficacy and safety of ponatinib using a novel, response-based, dose-reduction strategy in patients with CP-CML whose disease is resistant to ≥2 TKIs or who harbor T315I. To assess the dose-response relationship and the effect on the safety of ponatinib, we examined the outcomes of patients with CP-CML enrolled in PACE and OPTIC who received 45 mg/day of ponatinib. A propensity score analysis was used to evaluate AOEs across both trials. Survival rates and median time to achieve ≤1% *BCR::ABL1*^*IS*^ in OPTIC were similar or better than in PACE. The outcomes of patients with T315I mutations were robust in both trials. Patients in OPTIC had a lower exposure-adjusted incidence of AOEs compared with those in PACE. This analysis demonstrates that response-based dosing for ponatinib improves treatment tolerance and mitigates cardiovascular risk.

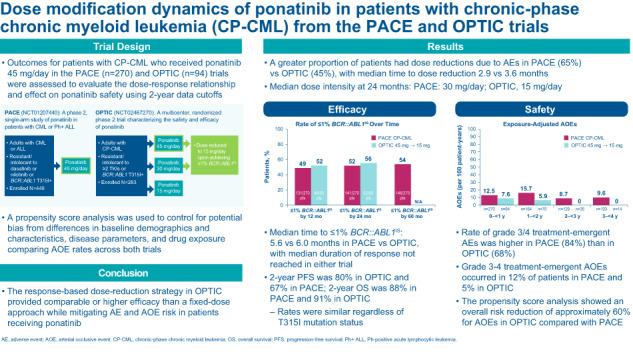

## Introduction

Chronic myeloid leukemia (CML) is a myeloproliferative neoplasm caused by constitutive activation of the BCR::ABL1 tyrosine kinase [1]. There are 3 defined phases of CML: chronic, accelerated, and blast phase. Chronic-phase CML (CP-CML) has a relatively mild and slow-growing presentation [2, 3], while accelerated and blast-phase CML are more diverse and aggressive [3]. Most patients are diagnosed in the chronic phase; without effective therapy, CP-CML can progress to later phases [1, 2]. Although tyrosine kinase inhibitors (TKIs) are highly effective in treating CP-CML, some patients fail to respond to or develop resistance to TKIs after an initial response [1, 4]. Relapsed/refractory (R/R) CML is associated with poor outcomes, with less durable responses after each successive line of therapy [4, 5]. TKI resistance is often the result of mutations in the *BCR::ABL* kinase domain, which impair TKI binding [1]. Among the known-*BCR::ABL* resistance mutations, the T315I “gatekeeper” mutation is resistant to imatinib and second-generation TKIs (dasatinib, nilotinib, and bosutinib) but is responsive to ponatinib, which is the only approved all known-BCR::ABL1 inhibitor third-generation TKI designed to potently inhibit BCR::ABL1 with or without any single resistance mutation, including T315I [6].

Dose-response relationships for TKIs are complex for several reasons. Imatinib was approved for CP-CML at a starting dose of 400 mg daily, reflecting that no maximum tolerated dose was established in the phase 1 study [7, 8], but a subsequent study suggested faster and deeper molecular responses at imatinib doses of 600 mg/day [9]. However, there is also evidence that some TKIs were initially approved at higher doses than needed to achieve substantial responses, resulting in increased adverse event (AE) rates, including cardiovascular events [7, 10]. Subsequent studies suggested that decreasing the doses of imatinib, dasatinib, nilotinib, and ponatinib maintained molecular response and improved safety [11–15]. Therefore, achieving the optimal balance between efficacy and toxicity in clinical trials is challenging. Further complications may arise as additional treatment options become available. Changes in patient populations, emergence of specific mutations upon treatment failure, and new therapies requiring different dose intensities may increase the risk of treatment-related toxicities [16–18].

Ponatinib has shown long-term responses of major cytogenic response (MCyR) or major molecular response (MMR) in patients with R/R CML or with the T315I *BCR::ABL1* mutation [4, 19]. In the phase 2 Ponatinib Ph+ ALL and CML Evaluation (PACE) trial, patients with CP-CML who failed to respond to prior second-generation TKI treatment demonstrated deep and durable molecular responses to 45 mg once daily doses of ponatinib [19]. However, after initiation of the study, arterial occlusive events (AOEs) emerged as a notable adverse event (AE), and dose adjustments were made for toxicity or based on recommendations from the US Food and Drug Administration (FDA) [20]. These observations led to the execution of the phase 2 Optimizing Ponatinib Treatment In CP-CML (OPTIC) trial to optimize ponatinib efficacy and safety. OPTIC randomized patients with CP-CML whose disease was resistant or intolerant to ≥2 BCR::ABL1 TKIs or with a T315I mutation to 1 of 3 starting doses of ponatinib, with protocol-driven dose reductions upon achievement of a prespecified response milestone (≤1% *BCR::ABL1*^*IS*^) [21]. Because the OPTIC trial did not have a control arm that maintained ponatinib at 45 mg once daily regardless of response, it did not provide a comparison of the efficacy and safety of response-based dosing with standard 45 mg daily dosing.

The aim of the current analysis is to compare outcomes in patients with CP-CML who started on a daily dose of 45 mg ponatinib in PACE, which did not have a response-based dosing strategy, and in OPTIC, which included prospective dose reductions after patients reached a response of ≤1% *BCR::ABL1*^*IS*^.

## Materials and methods

Detailed methods for the PACE and OPTIC trials have been previously described [19, 21]. Briefly, PACE (NCT01207440) was a phase 2, single-arm study of ponatinib in 449 patients with CML or Ph+ ALL whose disease was resistant or who were intolerant to dasatinib or nilotinib or who had the *BCR::ABL1* T315I mutation. Patients in PACE received ponatinib at a starting dose of 45 mg/day. The primary efficacy endpoint was MCyR by 12 months. The PACE study did not include a prospective response-based dose-reduction strategy; dose adjustments were made for toxicity or mandatory dose reduction based on the FDA’s regulatory guidelines after AOEs were identified. OPTIC (NCT02467270) is a multicenter, randomized phase 2 trial characterizing the safety and efficacy of ponatinib in 283 patients with CP-CML whose disease was resistant or intolerant to ≥2 TKIs or who harbored the *BCR::ABL1* T315I mutation [21]. Patients in OPTIC were randomized to receive a starting dose of 45 mg, 30 mg, or 15 mg ponatinib daily [21]. Patients in the 45-mg and 30-mg cohorts had their dose reduced to 15 mg daily upon achieving ≤1% *BCR::ABL1*^*IS*^, per study protocol [21]. As with PACE, dose adjustments in OPTIC were also made for toxicities. OPTIC had more stringent exclusion criteria for patients with cardiovascular risk factors including patients with uncontrolled hypertension, patients with any history of myocardial infarction, unstable angina, cerebrovascular accident, transient ischemic attack, or peripheral vascularization procedure, and patients with venous thromboembolism or congestive heart failure within 6 months of enrollment. This dosing dynamics analysis was conducted on the CP-CML cohort (*N* = 270) of PACE and 45-mg cohort (*N* = 94) of OPTIC using the 2-year data cutoffs for both studies. Each study population was analyzed separately for efficacy and safety. Categorical data are summarized by number and percentage of patients.

The PACE and OPTIC studies were conducted in accordance with the Declaration of Helsinki, US Food and Drug Administration–informed consent regulations, and International Conference on Harmonisation’s Good Clinical Practice guidelines. The protocols and amendments for both studies were approved by institutional review boards (IRCs) and ethics committees at participating centers. All patients provided written informed consent prior to enrollment in the PACE or OPTIC trials.

### Dosing

In PACE, proactive dose reductions were mandated in 2013, approximately 2 years after initiation of the last patient. Patients who achieved MCyR had doses reduced to 15 mg once daily and those without MCyR had doses reduced to 30 mg once daily unless benefit-risk analysis justified treatment with a higher dose. Dose reductions for AEs were permitted in PACE (to 30 or 15 mg). OPTIC was designed to incorporate a mandatory response-based dose-reduction strategy, as previously described [21]. Re-escalation of dose was permitted if response was lost, and dose reductions for AEs were also permitted (to a minimum of 10 mg) [21].

### Efficacy assessments

Response assessments included the percentage of patients achieving ≤1% *BCR::ABL1*^*IS*^, time to response, median duration of response (DOR), and median time on therapy. Molecular response was assessed in peripheral blood samples every 3 months by *BCR::ABL1*^*IS*^ measurement via quantitative real-time PCR of the *BCR::ABL1*^*IS*^ transcript in peripheral blood at a central molecular diagnostics laboratory, and results were reported to the participating investigator [4, 21]. Survival outcomes include progression-free survival (PFS), defined as the interval between first dose of ponatinib and disease progression (progression to accelerated-phase [AP] CML or blast-phase [BP] CML, loss of complete hematologic response [CHR] or MCyR, or doubling of white blood cell count to >20 000/mm^3^ on 2 occasions at least 4 weeks apart in patients without CHR) or death from any cause, and overall survival (OS), defined as the interval between the first dose of ponatinib and death from any cause [4, 21].

### Safety assessments

AEs were continuously assessed and graded according to the National Cancer Institute Common Terminology Criteria for Adverse Events, version 4.0 [4, 21]. In PACE, AOEs were retrospectively adjudicated. Briefly, an adjudication committee followed a predefined process in the adjudication charter. All suspected AOEs identified from the PACE AE data set were assessed using the charter definitions for myocardial infarction, heart failure attributed to an AOE (which may include coronary artery disease, arterial hypertension, cardiomyopathy, or myocardial infarction), hospitalization for unstable angina, stroke and other cerebrovascular events, and peripheral vascular disease. Events meeting these criteria were considered adjudicated AOEs (full details are reported in Januzzi et al, [20]). In OPTIC, AOEs were prospectively adjudicated [21]. Exposure-adjusted AOE rates were calculated as (number of first events in interval/total exposure for interval in patient-years) × 100.

### Analyses

The propensity score analysis was conducted to control for potential bias from differences in baseline characteristics when comparing the treatment emergent (TE)-AOE incidence rates in PACE and OPTIC. The propensity score calculation adjusted for 14 parameters including baseline characteristics (age, sex, race, ethnicity, geographic region, height, weight, Eastern Cooperative Oncology Group performance status, baseline systolic blood pressure, history of diabetes), disease parameters (time since diagnosis, mutation at baseline), and exposure (total dose, total number of days on drug).

## Results

### Patient baseline characteristics

Median follow-up was 57 months in PACE and 32 months in OPTIC [19, 21]. Only 2% of patients in PACE and 3% of patients in OPTIC had ≤1% *BCR::ABL1*^*IS*^ response at baseline (Table S1). Most patients in PACE (93%) and OPTIC (99%) received ≥2 prior TKIs; 60% of patients in PACE and 53% of patients in OPTIC received ≥3 prior TKIs; and most stopped any prior therapy due to resistance (PACE, 84%; OPTIC, 98%) [4, 21]. Nearly half of all patients (49% in PACE and 44% in OPTIC) had ≥1 *BCR::ABL1* kinase domain mutations, and a similar proportion of patients in each trial (24% in PACE and 27% in OPTIC) had the T315I mutation. Overall, patients were older in PACE (median age 60 years) than in OPTIC (median age 46 years). Although rates of vascular disorders and hypercholesterolemia as cardiovascular risk factors were higher in PACE than in OPTIC (44% and 24% in PACE versus 32% and 3% in OPTIC, respectively), other baseline characteristics were generally similar across PACE and OPTIC. The most common reasons for treatment discontinuation in PACE were AEs (13%) and lack of efficacy (clinical determination by the treating investigator)/progressive disease (11%) [4]. The most common reasons for treatment discontinuation in the 45-mg cohort of OPTIC were lack of efficacy/progressive disease (20%) and AEs (17%) [21].

### Dose reductions and dose intensity in PACE and OPTIC

**Fig. 1:**
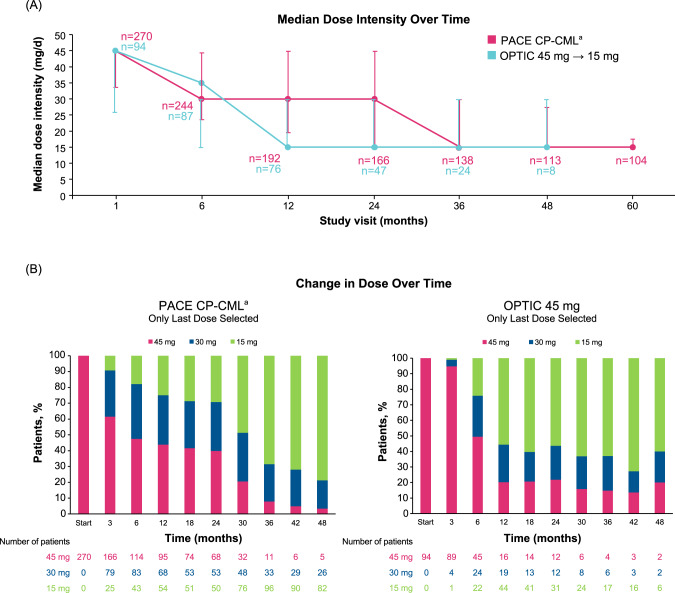
Ponatinib dose intensity and change in dose over time. **A** Line graph depicting median dose intensity over time. **B** Bar graph depicting change in dose over time with only the last dose selected at ≥2 months in (left) the PACE CP-CML cohort and in (right) the OPTIC 45-mg**→**15-mg cohort. ^a^PACE: Includes 3 patients with CP-CML who did not have a T315I mutation at study entry and were not resistant to dasatinib or nilotinib. CP-CML chronic-phase chronic myeloid leukemia.

A greater proportion of patients in PACE had their dose reduced due to AEs, 65% in PACE versus 45% in OPTIC, reflecting the design of the study. A similar percentage of patients had no dose reductions, 18% in PACE and 20% in OPTIC (Table 1). The overall median time to dose reduction was 2.9 months in PACE and 3.6 months in OPTIC; the median time to dose reduction for safety was 2.1 months in PACE and 6.3 months in OPTIC. The median dose intensity at 24 months was 30 mg/day for the PACE CP-CML cohort and 15 mg/day for the OPTIC 45-mg cohort. The median dose intensity decreased more rapidly over time in OPTIC than in PACE (Fig. 1A). As expected from the study design, patients in OPTIC experienced more rapid dose reductions over time compared with PACE (Fig. 1B).

**Table 1:** Efficacy, dose reductions, and dose intensity in PACE and OPTIC.

	PACE CP-CML 45 mg (*n* = 270)	OPTIC 45 mg→15 mg (*n* = 94)^a^
Median time to response (≤1% *BCR::ABL1*^*IS*^), mo	5.6	6
Median duration of response (≤1% *BCR::ABL1*^*IS*^), mo	NR	NR
Median time on therapy, mo	12.6	19.5
No dose reductions, *n* (%)	49 (18)	19 (20)
Median time to dose reduction, mo	2.9	3.6
Safety	2.1	6.3
Efficacy^b^	23.8	2.6

*AE* adverse event, *CP-CML* chronic-phase chronic myeloid leukemia, *FDA* US Food and Drug Administration, *mo* months, *NR* not reached.

^a^Efficacy was assessed in the intention-to-treat population (*N* = 276), which included 93 patients in the 45-mg cohort, 93 patients in the 30-mg cohort, and 90 patients in the 15-mg cohort.

^b^In PACE, these are dose reductions that were FDA mandated after 2013 for safety concerns [20]; in OPTIC, these are per protocol dose reductions implemented upon reaching ≤1% *BCR::ABL*^*IS*^ according to the study design.

### Efficacy outcomes

Response rates for patients who achieved ≤1% *BCR::ABL1*^*IS*^ by 24 months (Fig. S1) were 52% in PACE and 56% in OPTIC. Median time to ≤1% *BCR::ABL1*^*IS*^ was 5.6 months in PACE and 6.0 months in OPTIC. Median DOR was not reached in either trial. Overall, patients in OPTIC were on therapy longer than in PACE as median time on therapy was 12.6 months in PACE and 19.5 months in OPTIC (Table 1). Regardless of starting dose, median *BCR::ABL1*^*IS*^ transcript levels in the OPTIC patient population decreased substantially by Month 3 with the greatest decrease observed in the 45-mg cohort. Patients who received starting doses of 45 mg and 30 mg ponatinib and who had dose reductions to 15 mg also had a substantial decrease in *BCR::ABL1*^*IS*^ transcript levels by Month 3.

**Fig. 2:**
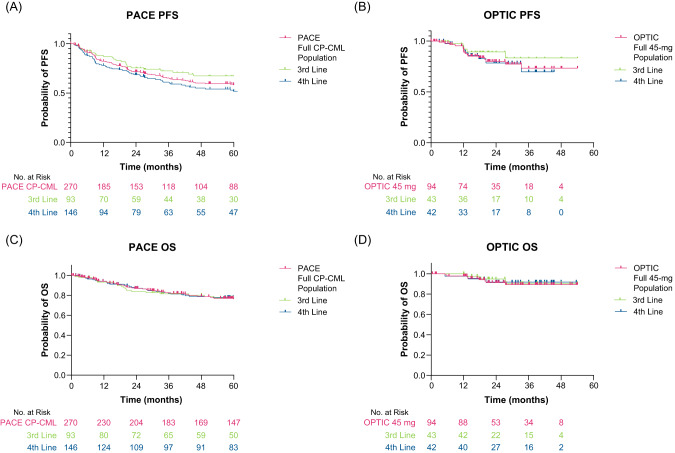
Progression-free survival and overall survival in PACE and OPTIC by line in therapy. Kaplan–Meier survival curves depicting **A** PACE PFS, **B** OPTIC PFS, **C** PACE OS, and **D** OPTIC OS. CP-CML chronic-phase chronic myeloid leukemia, OS overall survival, PFS progression-free survival.

The 2-year PFS was 80% in OPTIC and 67% in PACE; 2-year OS was similar in both trials, 88% in PACE and 91% in OPTIC. When broken down by ponatinib treatment in line of therapy, 2-year PFS was numerically higher in patients treated with ponatinib in the third-line versus patients treated with ponatinib in the fourth line for both PACE and OPTIC (Fig. 2A, B, respectively). However, 2-year OS was similar regardless of ponatinib line of therapy in both PACE and OPTIC (Fig. 2C, D, respectively). The 2-year PFS and OS rates were similar in patients with the T315I mutation compared with patients without the T315I mutation in both PACE and OPTIC (Fig. S2).

### Safety outcomes

The incidence of grade 3 or 4 TEAEs was higher in PACE (84%) than in OPTIC (68%). Exposure-adjusted AOEs were 12.5% in PACE and 7.6% in OPTIC at 0–<1 year and 15.7% in PACE and 5.9% in OPTIC from 1 to <2 years. Grade 3–4 TE-AOEs were 12% in PACE and 5% in OPTIC, and serious TE-AOEs were 15% in PACE and 4% in OPTIC (Fig. S3). The propensity score analysis, which accounted for a variety of factors including baseline risk factors, showed an overall risk reduction of approximately 60% for AOEs in OPTIC compared with PACE (Table 2).

**Table 2:** Propensity score analysis comparing AOE incidence in PACE and OPTIC.

Safety parameter	PACE CP-CML 45 mg (*n* = 270)	OPTIC 45 mg→15 mg (*n* = 94)
Patients with AOE	61	9
Unadjusted AOE rate (95% CI)	0.2230 (0.1733–0.2728)	0.0879 (0.0297–0.1461)
Adjusted AOE rate		
Odds ratio (95% CI)	—	0.3288 (0.1499–0.7212)
Relative risk (95% CI)	—	0.4066 (0.2060–0.8027)

*AOE* arterial occlusive event, *CI* confidence interval, *CP-CML* chronic-phase chronic myeloid leukemia.

## Discussion

The response-based dose-reduction strategy in OPTIC resulted in more rapid dose reductions, fewer dose reductions related to AEs, and longer median time on therapy compared with the initial fixed-dose strategy employed in PACE. Achievement of ≤1% *BCR::ABL1*^*IS*^ by 2 years and 2-year OS and PFS were higher in OPTIC than in PACE. The trend toward improved efficacy in OPTIC was observed even though OPTIC had more patients with treatment resistance at baseline than PACE (98% vs 84%, respectively). At 2 years, OPTIC had a notably lower incidence of grade 3 or 4 TEAEs, including AOEs, than PACE. After adjusting for differences in baseline characteristics, patients in OPTIC had an approximately 60% reduction in risk for AOEs compared with PACE, and AOE rates decreased with time on treatment [19, 21]. Differences in PACE and OPTIC cardiovascular exclusion criteria may be considered when interpreting the overall reduction in risk for AOEs. The median time to dose reduction for safety was 3 times longer in OPTIC than in PACE, and median time to dose reduction for efficacy was 9 times longer in PACE than in OPTIC. The results presented here support the finding that the response-based dose-reduction strategy (45→15 mg/day) provides comparable or better efficacy than a fixed-dose approach while mitigating the risk of AEs and AOEs in patients receiving ponatinib [21].

Since 2012, ponatinib has been approved as treatment for CP-CML, AP-CML, and BP-CML, and Ph+ ALL resistant or intolerant to TKIs and has a large clinical data set (*N* = 553) supporting its efficacy (46% CCyR by 12 months in PACE; 52% ≤1% *BCR::ABL1*^*I*S^ by 12 months in OPTIC) [4, 21] and safety in this highly resistant population [22, 23]. Although no direct comparisons are available, ponatinib appears to provide the highest probability of achieving ≤1% *BCR::ABL1*^*IS*^ and higher rates of OS compared with second-generation TKIs in the third-line treatment setting of patients with CP-CML with and without the T315I mutation [21, 24–27]. Ponatinib’s AOE signal was not initially identified during early clinical development [28]. Cardiovascular toxicities, including AOEs, have emerged as notable treatment-related AEs with long-term follow-up of most BCR::ABL1 TKIs [19, 29–31]; however, whether cardiovascular toxicity reflects an effect of the drug class or is TKI-specific is unknown. Studies have evaluated dose decreases from the initial approved dose and found better safety outcomes without compromising on efficacy [10, 15, 32]. Consideration of long-term safety data is imperative when optimizing dosing for oncology therapies. Asciminib, a Specifically Targeting the ABL Myristoyl Pocket (STAMP) kinase inhibitor, was approved by the FDA in 2021 for the treatment of patients with Ph+ CP-CML previously treated with ≥2 TKIs or with the T315I mutation [27, 33]. In the phase 3 ASCEMBL trial, with a median follow-up of 14.9 months, 49% of patients treated with asciminib 40 mg twice daily achieved ≤1% *BCR::ABL1*^*IS*^ [27]. The most common grade ≥3 AEs were thrombocytopenia (22%) and neutropenia (18%), and the AOE rate was 3.2% (5/156) [27]. While ponatinib and asciminib are both approved for patients with Ph+ CP-CML previously treated with ≥2 TKIs or with the T315I mutation, there are no head-to-head studies comparing efficacy and safety.

The OPTIC trial demonstrated an improved benefit to risk ratio of response-based ponatinib dosing in the third- and fourth-line setting [21]. Third-line ponatinib demonstrated higher response rates than other TKIs as well as better outcomes [25]. A systematic review on the efficacy of third-line TKI therapy estimated that ponatinib has a > 90% likelihood of providing a higher treatment response in the third-line setting than any of the 2G TKIs examined [25].

A retrospective analysis comparing the outcomes of patients with CML-CP treated with 2G TKIs or ponatinib as third-line TKI therapy found the 3-year PFS rate with ponatinib was 81% versus 60% with 2G-TKIs and the 3-year OS rate was 89% with ponatinib versus 81% with 2G TKIs; these survival outcomes were maintained after propensity matching [34]. Furthermore, third- or fourth-line ponatinib yielded response rates (≤1% *BCR::ABL1*^*IS*^: PACE, 46% by 12 months and 54% by ~5 years; OPTIC, 52% by 12 months and 56% by 24 months) [4, 19, 21] comparable or better than those reported for second-line bosutinib (≤1% *BCR::ABL1*^*IS*^, 46–50%) [35], suggesting ponatinib could be beneficial in the second-line setting. The current analysis demonstrated that long-term OS was favorable, regardless of whether patients received ponatinib in the third- or fourth-line setting. The ability of ponatinib to induce responses in patients harboring T315I mutations differentiates it from second-generation TKIs [1]. Patients with T315I mutation–positive disease who received 45 mg ponatinib had ≤1% *BCR::ABL1*^*IS*^ response rates of 60–70% [19, 21]. T315I mutations had little impact on survival, suggesting that dosing strategy does not affect ponatinib’s efficacy. Long-term outcomes with ponatinib by *BCR::ABL1* mutation status in this resistant CP-CML population will be discussed in future publications.

The limitations of this subanalysis include differences in study designs and patient populations for PACE and OPTIC, which preclude direct comparisons between the 2 trials. Additionally, PACE and OPTIC were open-label trials, and although the assessment of AOEs was prospective in both studies, the adjudication in PACE was retrospective [19, 21]. Head-to-head trials will need to be conducted to directly compare differences in outcomes between ponatinib and other treatments currently used in the treatment landscape.

In the absence of head-to-head trials, propensity score matching enables estimation and comparison of treatment effects and dosing strategies [36]. For example, propensity score–matched analyses have been used to compare efficacy and safety outcomes in patients with CP-CML receiving front-line imatinib, nilotinib, or dasatinib [37, 38]. Using this approach, we were able to perform comparisons of TE-AOE rates in PACE and OPTIC trial populations.

The response-based dose-reduction strategy in OPTIC resulted in more rapid dose reductions, fewer dose reductions related to AEs, and longer median time on therapy compared with PACE, while maintaining robust efficacy and survival outcomes. Results from this analysis demonstrate that response-adjusted dosing provides an approximately 60% reduced risk for AOEs. Findings from this analysis suggest that treatment with a response-based dose-reduction strategy provides comparable or better efficacy than a fixed-dose approach while mitigating risk of AEs and AOEs in patients receiving ponatinib therapy. Additionally, these data support the rationale to explore response-based dose-modification strategies for other BCR::ABL1 TKIs to reduce long-term toxicities and maintain efficacy.

### Supplementary information

Supplemental Material

Summary video

## Data Availability

The data sets, including the redacted study protocol, redacted statistical analysis plan, and individual participant data of the completed studies supporting the results reported in this article, will be made available within 3 months from initial request to researchers who provide a methodologically sound proposal. The data will be provided after de-identification, in compliance with applicable privacy laws, data protection, and requirements for consent and anonymization.
