# Correction: Dose modification dynamics of ponatinib in patients with chronic-phase chronic myeloid leukemia (CP-CML) from the PACE and OPTIC trials

**DOI:** 10.1038/s41375-024-02385-6

**Published:** 2024-09-02

**Authors:** Elias Jabbour, Jane Apperley, Jorge Cortes, Delphine Rea, Michael Deininger, Elisabetta Abruzzese, Charles Chuah, Daniel J. DeAngelo, Andreas Hochhaus, Jeffrey H. Lipton, Michael Mauro, Franck Nicolini, Javier Pinilla-Ibarz, Gianantonio Rosti, Philippe Rousselot, Neil P. Shah, Moshe Talpaz, Alexander Vorog, Xiaowei Ren, Hagop Kantarjian

**Affiliations:** 1 The University of Texas MD Anderson Cancer Center, Houston, TX USA; 2 Imperial College London, London, UK; 3 Georgia Cancer Center, Augusta, GA USA; 4 Hôpital Saint-Louis, Paris, France; 5 Versiti Blood Research Institute, Medical College of Wisconsin, Milwaukee, WI USA; 6 S. Eugenio Hospital, Tor Vergata University, Rome, Italy; 7 Singapore General Hospital, National Cancer Centre Singapore, Duke-NUS Medical School, Singapore, Singapore; 8 Dana-Farber Cancer Institute, Boston, MA USA; 9 Universitätsklinikum Jena, Jena, Germany; 10 Princess Margaret Cancer Centre, Toronto, ON Canada; 11 Memorial Sloan Kettering, New York, NY USA; 12 Centre Léon Bérard, Lyon, France; 13 H. Lee Moffitt Cancer Center & Research Institute, Tampa, FL USA; 14 IRST/IRCCS “Dino Amadori”, Meldola (FC), Italy; 15 Hospital Mignot University de Versailles Saint-Quentin-en-Yvelines, Paris, France; 16 University of California San Francisco, San Francisco, CA USA; 17 Comprehensive Cancer Center, University of Michigan, Ann Arbor, MI USA; 18 Takeda Development Center Americas, Inc., Lexington, MA USA

**Keywords:** Chronic myeloid leukaemia, Phase II trials

Correction to: *Leukemia* 10.1038/s41375-024-02159-0, published online 29 January 2024

Following publication of the article, the authors realized that several data points in Figure S3B and the accompanying manuscript text were incorrect. This oversight has been addressed and the corrected figure has been added.

1. Supplementary information, Figure S3B: The percentage of patients with grade 3-4 TE-AOEs from the PACE CP-CML group was inaccurately rounded to 12%; the correct percentage is 11%. In addition, we have corrected the error in the manuscript on page 477 in the Safety outcomes section. The revised statement is below:
  Grade 3-4 TE-AOEs were 11% in PACE and 5% in OPTIC, and serious TE-AOEs were 15% in PACE and 4% in OPTIC (Fig. S3).
2. Supplementary information, Figure S3B: The number of patients with serious TE-AOEs in the PACE CP-CML group was cited incorrectly as 9; the correct number of patients is 40.

The corrected figure S3B is now included in the supplementary information.

The original article has been corrected.

## Supplementary information

Supplementary information

