# Supplementary material for: Dose modification dynamics of ponatinib in patients with chronic-phase chronic myeloid leukemia (CP-CML) from the PACE and OPTIC trials
Source: Leukemia. 2024 Jan 29;38(3):475–81. doi: 10.1038/s41375-024-02159-0 (PMC10912029; doi:10.1038/s41375-024-02159-0)

# Supplementary Information

**Table S1. Demographics and baseline disease characteristics.**

| **Characteristic** | **PACE**  **CP-CML**  **45 mg**  **(*n* = 270)^a^** | **OPTIC**  **45 mg → 15 mg  (*n* = 94)** |
| --- | --- | --- |
| Median age, y | 60 | 46 |
| Male gender, n (%) | 144 (53) | 50 (53) |
| Median time since diagnosis, y | 7.0 | 5.5 |
| **Patients with CV risk factors, n (%)** |  |  |
| Hypercholesterolemia | 65 (24) | 3 (3) |
| Baseline BMI ≥30 kg/m^2^ | 73 (27) | 26 (28) |
| Diabetes mellitus | 32 (12) | 5 (5) |
| Vascular disorders | 119 (44) | 30 (32) |
| Arterial hypertension | 102 (38) | 29 (31) |
| Deep vein thrombosis | 4 (1.5) | 0 |
| Median CV risk factors (range) | 0 (0–3) | 0 (0–2) |
| **Concomitant medications, n (%)** |  |  |
| Statins | 64 (24) | 24 (26) |
| Acetylsalicylic acid | 87 (32) | 18 (19) |
| **Best response to last prior TKI^b^, n (%)** |  |  |
| CHR or worse | 171 (63) | 66 (70) |
| Better than CHR | 99 (37) | 28 (30) |
| ***BCR::ABL/ABL* ratio at baseline, n (%)** |  |  |
| ≤1% *BCR::ABL1^ISc^* | 6 (2) | 3 (3) |
| >1 to 10% *BCR::ABL1^IS^* | 54 (20) | 16 (17) |
| >10% *BCR::ABL1^IS^* | 200 (74) | 74 (79) |
| ***BCR::ABL1* mutation at baseline^d-g^, n (%)** |  |  |
| No mutation | 138 (51) | 51 (54) |
| T315I | 64 (24) | 25 (27) |
| Mutation other than T315I | 68 (25) | 16 (17) |
| ≥2 mutations detected | 29 (11) | 10 (11) |
| **Prior TKIs, n (%)** |  |  |
| 1 | 18 (6.7) | 1 (1.1) |
| ≥2 | 252 (93) | 93 (99) |
| ≥3 | 162 (60) | 50 (53) |
| **Resistance to prior therapy, n (%)** |  |  |
| Stopped prior therapy due to resistance, n (%) | 214 (84)^h^ | 92 (98) |
| Resistant to ≥2 prior TKIs | 242 (90) | 91 (97) |
| Resistant to ≥3 prior TKIs | 157 (58) | 49 (52) |
| Resistant to second-generation TKIs | 247 (91) | 91 (97) |

^a^ PACE: Includes 3 patients with CP-CML who were not T315I+ at study entry and not resistant to dasatinib or nilotinib. ^b^ OPTIC: Baseline response to last prior TKI is missing for 4 patients. ^c^ Patients with ≤1% *BCR::ABL1^IS^* at baseline were considered as a failure and excluded from the numerator for the efficacy analysis. ^d^ Sanger sequencing was used for mutation testing. ^e^ PACE: Four patients had an e1a2 variant, 4 had atypical transcripts, and 2 had missing data. ^f^ OPTIC: One patient in the 45-mg cohort does not have *BCR::ABL1*. ^g^ OPTIC: Two patients in the 45-mg cohort did not have any mutation testing performed at baseline. ^h^ Percentages were calculated according to the number of patients who received previous dasatinib or nilotinib: 256 patients with chronic-phase CML, 80 patients with accelerated phase CML, 61 patients with blast-phase CML, and 30 patients with Ph-positive ALL.

Abbreviations: BMI, body mass index; CHR, complete hematologic response; CP-CML, chronic-phase chronic myeloid leukemia; CV, cardiovascular; TKI, tyrosine kinase inhibitor; y, years.

**Figure S1. Efficacy outcomes in PACE and OPTIC by 12, 24, and 60 months.** Bar graph depicting ≤1% *BCR::ABL1^IS^* rates by 12, 24, and 60 months. Percentages are above each bar. N/A, not available.


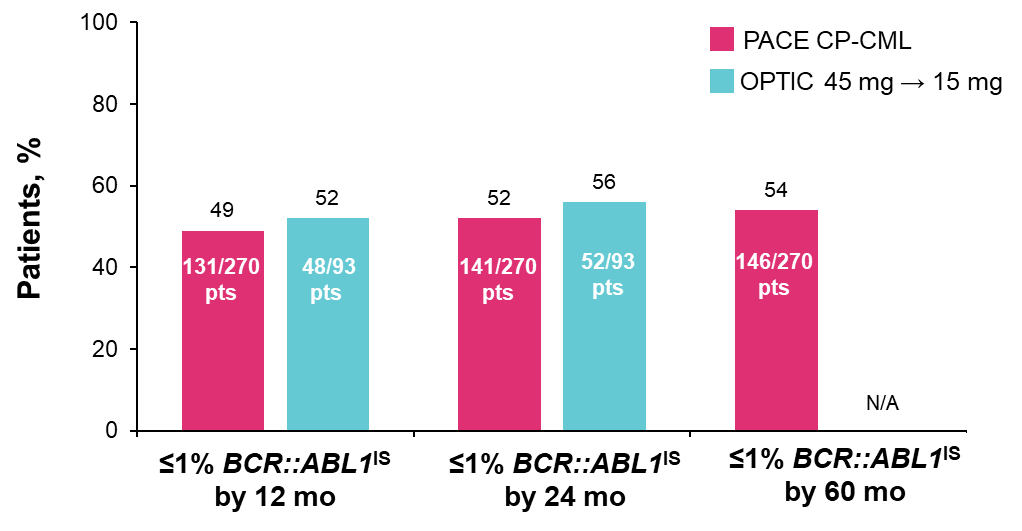


## **Figure S2. Progression-free survival and overall survival in PACE and OPTIC by mutation.** Kaplan-Meier survival curves depicting (A) PACE PFS, (B) OPTIC PFS, (C) PACE OS, and (D) OPTIC OS. Number at risk is indicated at the bottom of the graph for each time point. Two-year survival for the overall population is indicated on each graph in red.

#
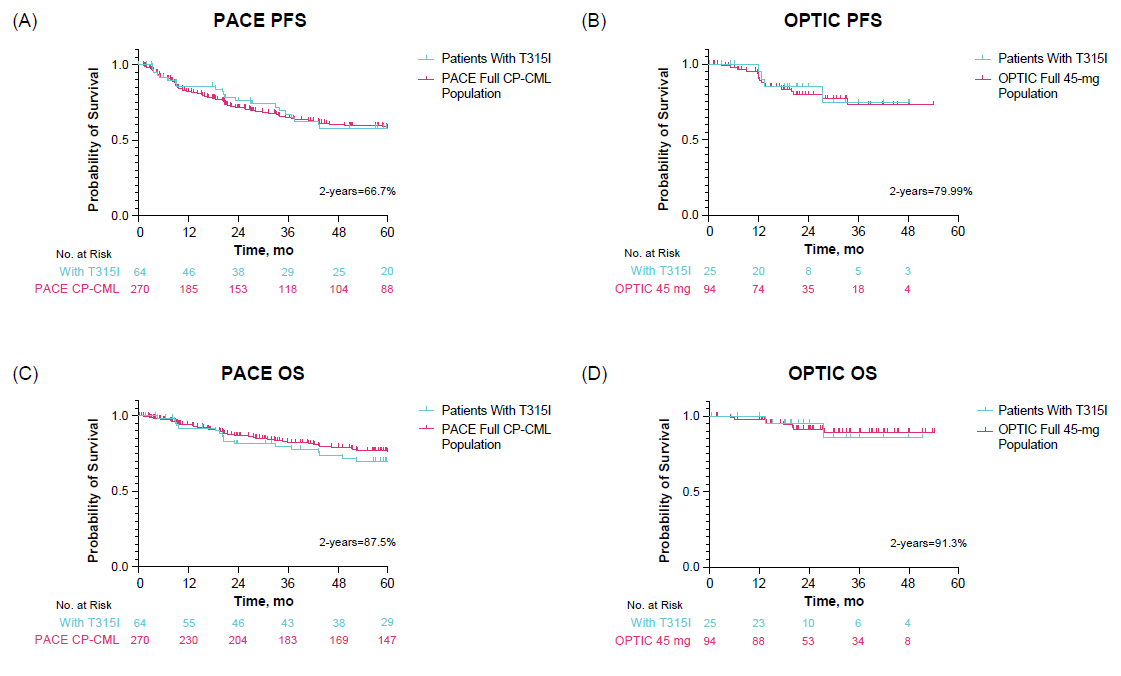


**Figure S3. (A) Exposure-adjusted AOEs and (B) Grade 3–4 and serious AOE rates in PACE and OPTIC.** (A) Exposure-adjusted AOEs. Cohort numbers are at the top of each graph for each time point. (B) Left portion of the graph is the PACE CP-CML cohort, and right portion of the graph is OPTIC 45 mg **→** 15 mg cohort. Cohort numbers are at the top of each graph.

## (A) **Exposure-Adjusted AOEs**


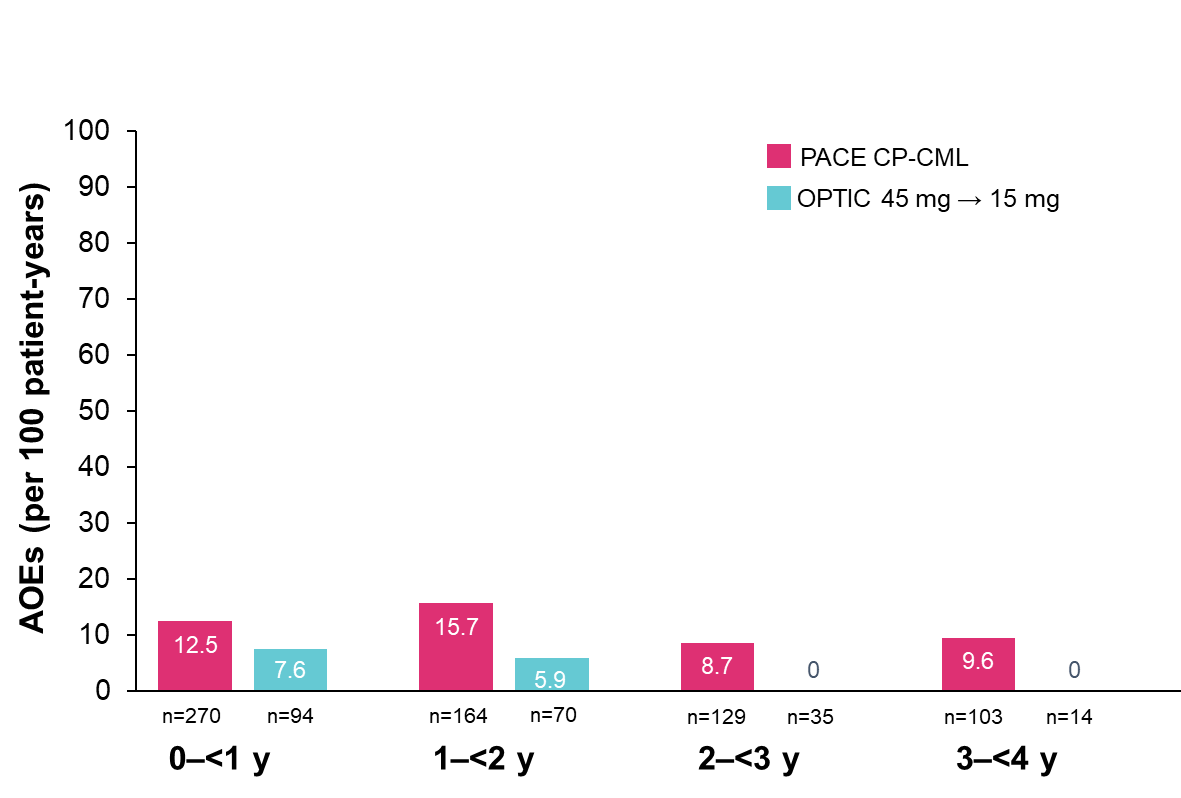


#### (B) **Grade 3–4 and Serious AOE Rates in PACE and OPTIC**


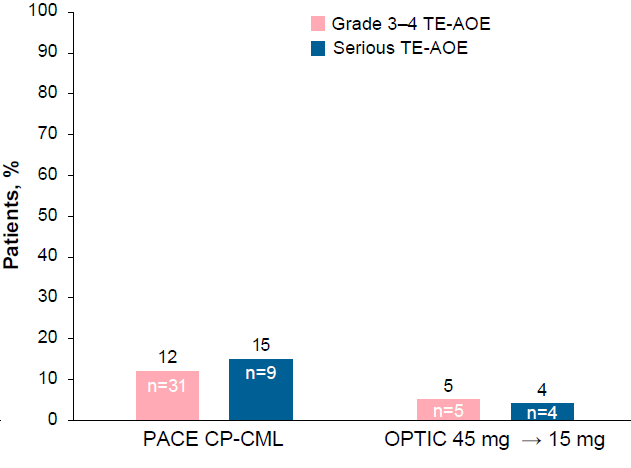

Supplement: Supplementary file 1 — Supplemental Material [file 41375_2024_2159_MOESM1_ESM.docx]
